# Preparation and Characterization of All-Biomass Soy Protein Isolate-Based Films Enhanced by Epoxy Castor Oil Acid Sodium and Hydroxypropyl Cellulose

**DOI:** 10.3390/ma9030193

**Published:** 2016-03-15

**Authors:** La Wang, Jianzhang Li, Shifeng Zhang, Junyou Shi

**Affiliations:** 1 MOE Key Laboratory of Wooden Material Science and Application, Beijing Forestry University, Beijing 100083, China; bjfuwl@163.com (L.W.); lijzh@bjfu.edu.cn (J.L.); 2 College of Forestry, Beihua University, Jilin 132013, China

**Keywords:** soy protein isolate, films, epoxy, all-biomass, castor oil

## Abstract

All-biomass soy protein-based films were prepared using soy protein isolate (SPI), glycerol, hydroxypropyl cellulose (HPC) and epoxy castor oil acid sodium (ECOS). The effect of the incorporated HPC and ECOS on the properties of the SPI film was investigated. The experimental results showed that the tensile strength of the resultant films increased from 2.84 MPa (control) to 4.04 MPa and the elongation at break increased by 22.7% when the SPI was modified with 2% HPC and 10% ECOS. The increased tensile strength resulted from the reaction between the ECOS and SPI, which was confirmed by attenuated total reflectance-Fourier transform infrared spectroscopy (ATR-FTIR), scanning electron microscopy (SEM) and X-ray diffraction analysis (XRD). It was found that ECOS and HPC effectively improved the performance of SPI-based films, which can provide a new method for preparing environmentally-friendly polymer films for a number of commercial applications.

## 1. Introduction

Plastics with low cost, good mechanical properties, durability, disposability and chemical resistance bring great convenience to our daily lives [1]. However, with the increased concerns about dwindling petroleum reserves and environmental problems, biodegradable plastics have attracted intense interest and will provide a promising alternative to petroleum-based plastics [2,3,4,5]. Over the past few decades, proteins have been extensively investigated as replacements for their petroleum-based counterparts, due to their low cost, renewability, biocompatibility and biodegradability [6]. The protein content of soy protein isolates (SPI) is higher than other protein products, which facilitates film-forming capabilities [7,8]. Protein plays a multifunctional role in providing tightness to liquid, barriers to gases, and bonding layers for making films or coating [9,10]. Furthermore, protein-based films and coatings are non-toxic, degradable, constraining enzymatic browning of fresh-cut products and inhibiting polyphenoloxidase adventitious in foods [11,12]. However, the extensive use of SPI films has been limited because of their low tensile strength, poor water resistance and moisture barrier properties [13,14,15]. Much effort has been expended to improve these properties through physical, chemical and combination modifications of SPI materials [16,17,18,19,20,21]. Generally, the increased tensile strength of modified SPI-based films has consistently resulted in decreased flexibility due to the restricted molecular motion of the SPI main polymer chain [19,22].

The natural polymer cellulose has been extensively employed in practical products, because of its chemical stability, biocompatibility, biodegradability and sustainability. Modified cellulose and its derivatives are more conducive to enhance the properties of materials than the raw cellulose [23]. Small amounts of hydroxypropyl cellulose (HPC) are very compatible with SPI, but microphase separation appears between SPI and HPC as the quantity of HPC in the mixture is increased, which will result in inferior mechanical properties [20].

Castor oil has attracted much attention as a polymer modified in the past few decades due to its environmentally-compatibility, biodegradability and abundance [24]. The alkene double bond in natural oils together with abundant hydroxyl and ester groups in the molecular chains, provide these raw materials with ample reaction sites for esterification, hydrogenation, alcoholysis, interesterification and epoxidation. In general, epoxidation has been found to be one of the main methods for chemical modification of vegetable oils [25]. The epoxidized oil was widely used in a variety of films and coatings for improving their properties [26]. Benaniba *et al.* [27] reported that the thermal and mechanical behavior of polyvinyl chloride (PVC) was enhanced by addition of epoxidized oil. Moreover, the flexibility of polyurethane-molded plastic films could be improved after adding epoxidized oil [28]. Previous research has demonstrated that the thermal stability and water resistance of poly (vinyl alcohol) (PVA) films could be greatly enhanced by the introduction of epoxidized castor oil (ECO) [29]. It has also been demonstrated that the epoxy groups reacted readily with the amino groups in soy protein and improved the water resistance of resulting soy protein-based composite [30,31]. Epoxidized soybean oil (ESO) cured with terpene-based acid anhydride showed a higher glass transition temperature and tensile strength than other modified ESOs [32]. 

In this reported study, the epoxidized castor oil acid sodium (ECOS) was synthesized, saponified and employed to enhance the performance of the SPI-based films. Several SPI/HPC films modified by ECOS were prepared by casting methods and alkenyl succinic anhydrides (ASA) was used as the polymerization catalyst [33]. The effect of the ECOS addition on the thermal stability of the modified SPI films was examined. The tensile strength (TS) and elongation at break (EB) of the SPI-based films were also evaluated.

## 2. Experimental

### 2.1. Materials

SPI with 2.0% moisture content and 88.0%–95.0% protein content was kindly provided by Yuwang Ecological Food Industry Co., Ltd. (Shandong, China). The glycerol and analytical reagent acetic acid were obtained from Beijing Chemical Works Co., Ltd. (Beijing, China). HPC (M_W_ = 100,000) was obtained from Alfa Aesar Co., Ltd. (Shanghai, China). The castor oil was obtained from Sinopharm Chemical Reagent Co., Ltd. (Shanghai, China). The hydrogen peroxide with a concentration of 30% was obtained from Xilong Chemical Company Co., Ltd. (Guangdong, China). Other chemical reactants of analytical grade were purchased from Beijing Chemical Reagents Co., Ltd. (Beijing, China) and used as received.

### 2.2. Synthesis of ECO

**Figure 1:**
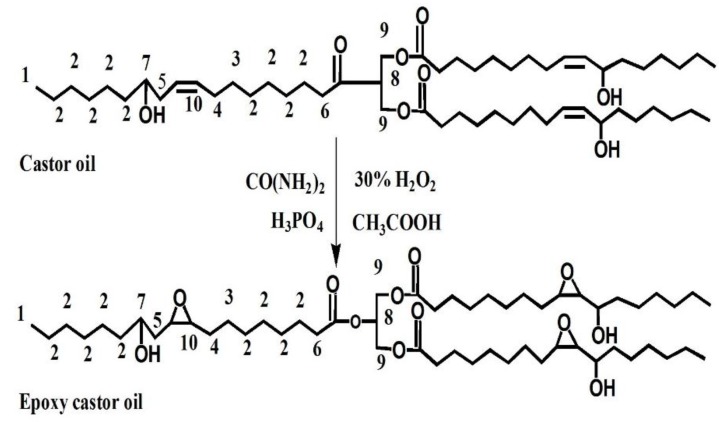
Synthesis schematic of ECO.

The epoxidation of castor oil was conducted according to the following procedure: the 60 g castor oil was treated with 12 g CH_3_COOH and the 4 g urea was used as stabilized agent. The 48 g H_2_O_2_ together with 4 g H_3_PO_4_ was added to this mixture stirred for 60 min at 35 °C. This chemical reaction was then incubated at 60 °C for 150 min. The resultant product was washed three times with 10% K_2_CO_3_ solution and deionized H_2_O, and then the oil layer was separated using a rotary evaporator [34]. Finally, ECOS was obtained by the saponification reaction of ECO and sodium hydroxide. The schematic diagram of epoxidized castor oil is shown in Figure 1.

### 2.3. Preparation of SPI films

Various SPI solutions were prepared according to the following procedure. A set of SPI and glycerol were firstly added to deionized water and mixed homogeneously. The mixtures were heated in a water bath for 30 min at 85 °C and the solution pH was maintained at 8. Various amounts of HPC were added to the SPI solution, mixed uniformly and ultrasound-treated (750 W, 20 KHz, VC-750, Sonics and Materials, Newton, CT, USA) for 5 min to dissolve HPC and remove trapped air bubbles. Then, ECOS and ASA were added to Samples E and F and stirred uniformly. A film-casting solution resulted from this reaction (40 mL) was poured into leveled Teflon plates, dried at 45 °C for 20 h and peeled off the substrate for later use. The detailed formulations of all the produced films are listed in Table 1.

### 2.4. ^1^H Nuclear Magnetic Resonance (NMR) 

^1^H NMR spectra of the produced films were obtained using a JEOL DELTA2 600 MHz FX-1000 spectrometer (JEOL Ltd., Tokyo, Japan) employing deuterated chloroform (CDCl_3_) and tetramethylsilane as solvent and internal standard, respectively.

### 2.5. Film Characterization

#### 2.5.1. Equilibrium Treatment

Films were equilibrated with 50% relative humidity at 25 ± 1 °C for 48 h in a desiccator using saturated salt solutions of K_2_CO_3_ prior to testing [17]. 

#### 2.5.2. Film Thickness

Film thicknesses were measured using a digital micrometer (Measuring & Cutting Tool Works Co., Ltd., Shanghai, China)with an accuracy of 0.001 mm. Measurements were taken at five random locations on each film. The average thickness value of each film was calculated for later use in the mechanical properties measurements.

#### 2.5.3. Mechanical Properties

The tensile strength of films was determined using a universal testing machine (INSTRON 3365, Norwood, MA, USA) at room temperature at a crosshead speed of 50 mm·min^−1^. Five replicates for each film were conducted and the average value was reported.

#### 2.5.4. X-ray Diffraction Analysis (XRD)

X-ray diffraction tests were conducted using a D8 Advance diffractrometer (Bruker AXS, Karlsruhe, Germany) with a Cu-Kα source in continuous scanning mode, operating at 45 kV and 30 mA ranged from 5° to 60° (2θ) at 2°·min^−1^. The crystal state was calculated using associated accessory software (DIFFRAC.EVA; V3.1).

#### 2.5.5. Attenuated Total Reflectance-Fourier Transform Infrared Spectroscopy (ATR-FTIR)

The attenuated total reflectance (ATR) Fourier transform infrared spectroscopy (FTIR) was used to examine the chemical structures of the films. A Nicolet 6700 FTIR spectrometer (Thermo Scientific, Madison, WI, USA) with an ATR accessory was employed for these tests at the range of 600 and 4000 cm^−1^ with 4 cm^−1^ resolution for 32 scans. 

#### 2.5.6. Thermogravimetric Analysis (TGA)

The thermal stabilities of films were examined using a Q50 TGA device (TA Instruments, New Castle, DE, USA) with a heating rate of 10 °C·min^−1^ from room temperature to 600 °C under constant N_2_ stream (100 mL·min^−1^) to avoid thermo-oxidative reactions. The maximum degradation rate was calculated as the mass (%) at peak temperature divided by the peak temperature.

#### 2.5.7. Scanning Electron Microscopy (SEM)

An S-3400N Hitachi scanning electron microscope (SEM, Hitachi, Tokyo, Japan) with an accelerating voltage of 20 kV and a magnification of 1600× was used to observe the cross sectional morphologies of the films. Prior to the observations, the specimens were sputter-coated with gold to avoid charging under the electron beam. 

#### 2.5.8. Statistical Analysis

The analysis of variance (ANOVA) was used to evaluate the significance in the difference between means, whichwas considered as significant difference when *P* < 0.05.

## 3. Results and Discussion

### 3.1. Synthesis of ECO

**Figure 2:**
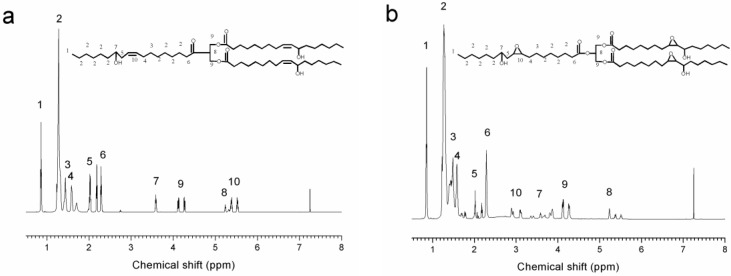
^1^H NMR spectra of (**a**) castor oil; and (**b**) ECO.

^1^H NMR spectra of castor oil and ECO are shown in Figure 2. The chemical shifts at *δ* = 0.8 ppm (peak 1) and *δ* = 4.2–4.3 ppm (peak 9) can be assigned to methyl protons and methylene protons of glycerol, respectively. The peak at *δ* = 5.4–5.6 ppm corresponds to the double bond protons -CH=CH- of castor oil fatty acid (peak 10, Figure 2a), which was consistent with that reported in the literature [35]. Compared to Figure 2a, the presented chemical shift at *δ* = 2.9–3.1 ppm (peak 10, Figure 2b), can be assigned to the epoxy groups of ECO (Figure 2b). These results indicated that C=C double bond was successfully oxidized to an epoxy bond. However, the peak at *δ* = 5.4–5.6 ppm greatly decreased but did not disappear, indicating that the epoxidation reaction was incomplete. This was due to the steric hindrance from double bonds located in the middle of molecule chains, which would affect the epoxidation efficiency [36].

### 3.2. ATR-FTIR Spectra of the SPI-Based Films

**Figure 3:**
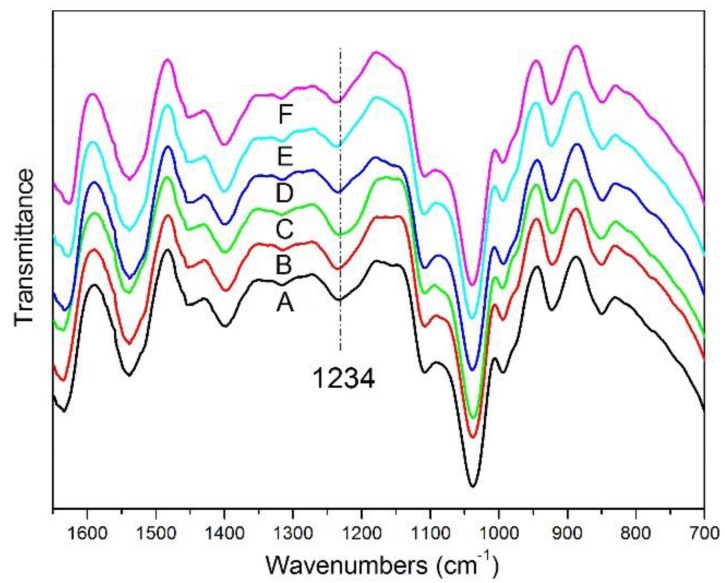
ATR-FTIR spectra of SPI-based films: (**A**) the control, (**B**) add 1% HPC, (**C**) add 2% HPC, (**D**) add 5% HPC, (**E**) add 2% HPC and 10% ECOS, (**F**) add 2% HPC, 10% ECOS and alkenyl succinic anhydrides (ASA).

The ATR-FTIR spectra of the experimental films are shown in Figure 3. The peaks at 1634 cm^−1^, 1538 cm^−1^ and 1234 cm^−1^ correspond to amide I (C=O stretching), amide II (N-H bending) and amide III (C-N and N-H stretching) of SPI, which was consistent with the previous report [37]. Comparing films A and F, all peaks intension of the films B, C and D was not significantly changed because no formed covalent bond existed between HPC and SPI [20]. It appeared that the peak intensities at 1234 cm^−1^ were corresponding to the decreased free amino groups in films E and F, which was probably a result of the reaction between epoxy groups of ECOS and amino groups in the SPI, corresponding to the Lei’s results [38]. 

### 3.3. Thermal Properties of the SPI-Based Films

**Figure 4:**
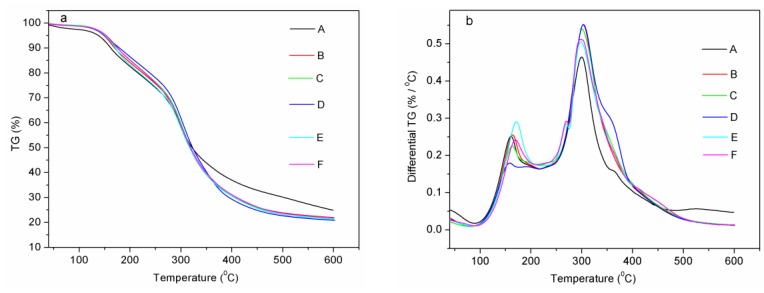
TG (**a**); and differential TG (**b**) patterns of the films: (**A**) the control, (**B**) add 1% HPC, (**C**) add 2% HPC, (**D**) add 5% HPC, (**E**) add 2% HPC and 10% ECOS, (**F**) add 2% HPC, 10% ECOS and alkenyl succinic anhydrides (ASA).

The weight loss and differential thermogravimetric (TG) curves of the SPI-based films were recorded from 40 to 600 °C and are shown in Figure 4. The results for the TGA thermal degradation of the SPI-based films are shown in Table 2. The weight loss range from 120 to 200 °C was related primarily to the evaporation of glycerol as the first decomposition Stage and the weight loss from 250 to 350 °C was caused by the thermal degradation of soy proteins at the second Stage [31]. The initial degradation temperature of the first Stage for films B and C was not significantly different, when compared with that of the pure protein film (film A). The first Stage degradation was contributed by the evaporation of glycerol, while the small amount of HPC would not affect the hydrogen bonding between glycerol and SPI [20]. However, the degradation temperature for film D in the first Stage was greatly decreased, owing to the formation of more paths for glycerol evaporation as a result of the HPC aggregation [39]. The temperatures of the initial degradation and maximum degradation rate in the first Stage for films E and F both increased due to the formed hydrogen bonds between ECOS and glycerol. The initial degradation temperature in the second Stage of films B, C and D were not significantly changed, compared with the pure protein film (film A), which was resulted from the physical crosslinking between HPC and SPI. In addition, the initial degradation temperature in the second Stage for films E and F increased significantly, confirming that the reaction occurred between epoxy groups of ECOS and amino groups in SPI.

### 3.4. Crystalline Properties of the SPI-Based Films

**Figure 5:**
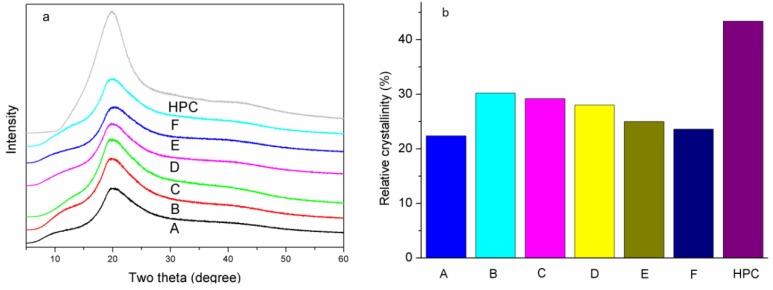
The XRD patterns (**a**); and relative crystallinity (**b**) of the films and HPC: (**A**) the control, (**B**) add 1% HPC, (**C**) add 2% HPC, (**D**) add 5% HPC, (**E**) add 2% HPC and 10% ECOS, (**F**) add 2% HPC, 10% ECOS and alkenyl succinic anhydrides (ASA).

The XRD diffraction patterns of the SPI-based films and HPC are shown in Figure 5a. Their relative crystallinities were calculated and are presented in Figure 5b. The XRD Peaks at 9.6° and 20.5° for film A corresponded to 7 s and 11 s globulins of the soy protein [40]. When the highly crystalline HPC was introduced into the SPI matrix, the relative crystallinity of films B, C and D increased, corresponding to the Zhou’s study [20]. Compared with film B, the relative crystallinity of film C decreased as a result of the increasing of molecular inter-atomic forces with the increase in hydrogen bonding formed between HPC and SPI [41]. This bonding restricted the SPI molecular rearrangement and resulted in decreased crystallinity and the relative crystallinity of film D decreased. When ECOS and ASA were added, the relative crystallinity of films E and F continued to decrease but was still higher than that of film A. These results confirmed the reaction between the epoxy groups and amino groups, which restricted the molecular motion of the SPI and ECOS, decreasing their crystallinity. 

### 3.5. Micromorphology of the SPI-Based Films

**Figure 6:**
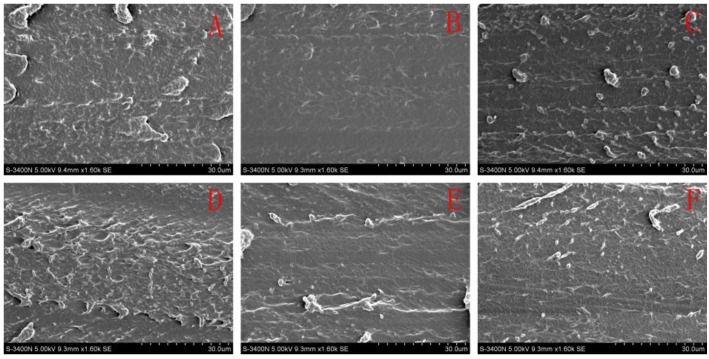
SEM micrographs showed cross-sections of the six films. Magnification: 1600. (**A**) the control; (**B**) add 1% HPC; (**C**) add 2% HPC; (**D**) add 5% HPC; (**E**) add 2% HPC and 10% ECOS; (**F**) add 2% HPC, 10% ECOS and alkenyl succinic anhydrides (ASA).

The cross-section morphological structure of the experimental films was observed by SEM as shown in Figure 6. Obviously, the surface of film A was rough and contained small pleats, which was consistent with the results of our previous studies [17]. The fractured surface of films B and C were smoother than film A, indicating that the small amount of HPC in SPI-based films was complete compatibility with the SPI matrix, thus forming a smoother surface [20]. When the amount of HPC was further increased, the cross-section of film D was rougher than the control (film A) because of the partially aggregated HPC. When ECOS was incorporated, the fractured surfaces of films E and F were smoother than film C, as a result of the reaction between ECOS and SPI [17].

### 3.6. Physical and Mechanical Properties of the SPI-Based Films

**Figure 7:**
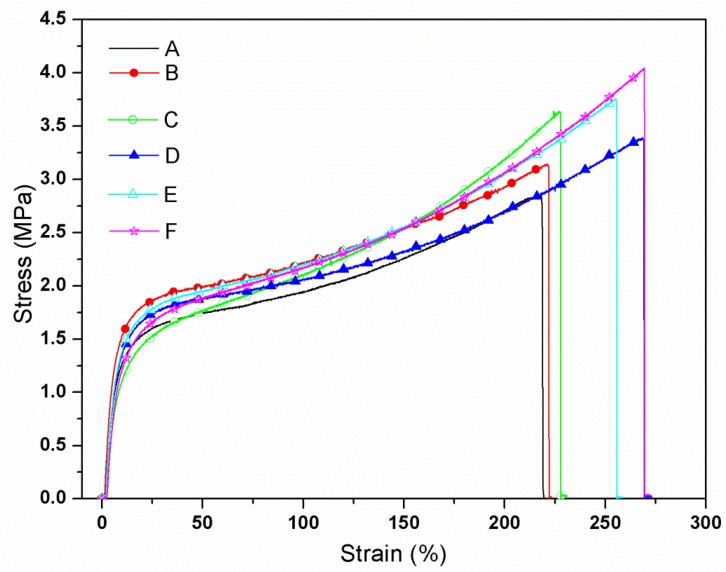
The stress-strain curves of the films. (**A**) the control, (**B**) add 1% HPC, (**C**) add 2% HPC, (**D**) add 5% HPC, (**E**) add 2% HPC and 10% ECOS, (**F**) add 2% HPC, 10% ECOS and alkenyl succinic anhydrides (ASA).

The tensile test results on properties of the experimental films are summarized in Table 3 and the strain-stress curves of the films are shown in Figure 7. The stress continuously increased with the increasing strain until brook without necking, indicating some isotropy in the film [42]. After the incorporation of HPC, the TS of the blended films increased and reached a maximum value in film C and then decreased (film D) with further increases in the HPC content. The decrease of TS probably resulted from the stress concentration in the blended film caused by the aggregation of HPC, which was consistent with the test results previously mentioned. Compared with film C, when ECOS and ASA were introduced, TS of the modified films (films E and F) were continuously enhanced, illustrating that ECOS successfully reacted with SPI.

EB of the films (films B and C) was not significantly altered in comparison to the control film (film A) as shown in Table 3. This can be reasoned that the HPC addition amount was too little in comparison to the SPI matrix [20]. When adding 5% HPC, EB of film D increased as a result of the partial aggregated HPC, which allowed the free motion of the SPI molecule. EB of the films (film E and F) increased as a result of the hydrogen bonding between ECOS and SPI and the long-chains of ECOS [43,44].

## 4. Conclusions

In this study, all-biomass SPI-based films were successfully prepared. The TS and flexibility properties of the protein-based films increased with the addition of HPC. XRD results indicated that HPC was completely compatible with the SPI matrix. ATR-FTIR results indicated that the opening ring reaction between epoxy groups of ECOS and amino groups of SPI. Compared with the control film, TS and EB of the film prepared from SPI, HPC, ECOS and ASA synchronously increased by 42.3% and 22.7%, respectively. 

## Figures and Tables

**Table 1:** The formulations of films A–F.

Sample	SPI (g)	Glycerol (g)	Water (g)	HPC (g)	ECOS (g)	ASA (g)
A	5	2.5	95	–	–	–
B	5	2.5	95	0.05	–	–
C	5	2.5	95	0.1	–	–
D	5	2.5	95	0.25	–	–
E	5	2.5	95	0.1	0.5	–
F	5	2.5	95	0.1	0.5	0.005

**Table 2:** Thermogravimetric analysis (TGA) parameters of the thermal degradation of the soy protein isolate (SPI)-based films.

Films	*T_i1_* (°C)	*T_max1_* (°C)	*T_i2_* (°C)	*T_max2_* (°C)
A ^1^	131.20	159.44	270.73	299.92
B ^2^	132.12	162.56	272.73	302.88
C ^3^	130.38	161.50	272.20	301.07
D ^4^	126.80	154.96	272.86	302.12
E ^5^	137.52	171.11	284.56	299.36
F ^6^	137.54	167.53	283.54	297.45

Note: *T_i_*, initial temperature of degradation; *T_max_*, temperature at maximum degradation rate: ^1^ the control; ^2^ add 1% HPC; ^3^ add 2% HPC; ^4^ add 5% HPC; ^5^ add 2% HPC and 10% ECOS; ^6^ add 2% HPC, 10% ECOS and alkenyl succinic anhydrides (ASA).

**Table 3:** The thickness, tensile strength (TS) and elongation at break (EB) of different SPI-based films.

Films	TS (MPa)	EB (%)	Thickness (mm)
	Average (SD)	Average (SD)	Average (SD)
A ^1^	2.84 (0.140) a	220.4 (4.0) a	0.239 (0.006) a
B ^2^	3.17 (0.132) b	221.8 (22.7) a	0.231 (0.021) a
C ^3^	3.63 (0.133) c	227.9 (8.8) ab	0.233 (0.017) a
D ^4^	3.38 (0.175) bd	271.8 (18.3) c	0.263 (0.009) b
E ^5^	3.75 (0.058) ce	257.3 (25.5) bc	0.207 (0.008) c
F ^6^	4.04 (0.171) f	270.4 (11.3) c	0.244 (0.015) d

Note: a,b,c,d Different letters in the same column indicate significant differences (*p* < 0.05); ^1^ the control; ^2^ add 1% HPC; ^3^ add 2% HPC; ^4^ add 5% HPC; ^5^ add 2% HPC and 10% ECOS; ^6^ add 2% HPC, 10% ECOS and alkenyl succinic anhydrides (ASA).
